# Supplementary material for: On the Uses and Abuses of Regression Models: A Call for Reform of Statistical Practice and Teaching
Source: Stat Med. 2025 Jun 24;44(13-14):e10244. doi: 10.1002/sim.10244 (PMC12186762; doi:10.1002/sim.10244)
Supplement: Supplementary file 1 — Data S1: Supporting Information. [file SIM-44-0-s001.docx]

**On the uses and abuses of regression models:
a call for reform of statistical practice and teaching**

John B. Carlin & Margarita Moreno-Betancur

Murdoch Children’s Research Institute & The University of Melbourne

***Supplementary Material***

**Appendix 1**

Additional details of Example 1 (kidney lengths in children with urinary tract infection, with and without pyelonephritis).

These data are publicly available here: https://doi.org/10.26188/26973436.v1.

**Figure A1.1**

Scatter plot of kidney length vs age for the two groups (red triangle = infected (pyelonephritis), blue circle = uninfected), with superimposed cubic regression lines (imposing constant group mean difference). This figure reproduces Figure 1 from the main text using colour and a better choice of plotting symbols to provide a clearer visualisation. Dashed vertical lines and grey bars also show the crude mean differences by five age groups (0-6 months, 6-12 months, 12-24 months, 24-36 months, 36-60 months)

**Table A1.1**

Summary statistics on kidney length by age group, for the infected and uninfected groups.

|  | Uninfected | | Infected | |  |  |
| --- | --- | --- | --- | --- | --- | --- |
| Age group | *n* | mean | *n* | mean | difference in means | pooled SD, *s* |
| 0-6 | 104 | 54.4 | 42 | 58.8 | 4.42 | 5.68 |
| 6-12 | 42 | 60.0 | 22 | 65.0 | 5.00 | 7.27 |
| 12-24 | 25 | 67.3 | 17 | 68.3 | 0.96 | 7.28 |
| 24-36 | 32 | 72.0 | 11 | 79.4 | 7.36 | 7.71 |
| 36-60 | 48 | 75.0 | 7 | 76.7 | 1.74 | 4.71 |

**Table A1.2**

Estimates of the difference in mean kidney length between infected and uninfected patients, obtained by methods that make increasingly strong modelling assumptions.

| Method | Estimate of mean difference | Estimated SE |
| --- | --- | --- |
| Simple difference of overall means  (equivalent to univariate regression on group indicator) | 2.56 | 1.20 |
| Simple average difference of means across 5 age groups | 3.90 | 0.91 |
| Weighted* average difference of means across 5 age groups | 3.95 | 0.74 |
| Regression-based estimate with adjustment for age groups using 4 indicator variables | 4.14 | 0.73 |
| Regression-based estimate with adjustment for age using cubic polynomial in continuous age | 4.13 | 0.71 |

*weights = inverse variance of age-group-specific mean differences

**Appendix 2**

**A brief review of the use of regression methods in contemporary medical research**

John B. Carlin & Margarita Moreno-Betancur

Murdoch Children’s Research Institute & The University of Melbourne

30-Aug-2023

**Objective**

The review aimed, for a series of articles representing “mainstream” clinical research published in three journals in June 2022, to:

1. Determine whether regression analysis was used,
2. Examine whether regression was used for a clear purpose, and if so for what type of purpose (descriptive, predictive, causal),
3. For those papers in which regression analysis was used, assess whether key misuses of regression were apparent.

**Methods**

The following three journals were identified from a listing of the top 20 “most influential medical journals” (Jemielniak et al, *J Med Internet Res.* 2019), to represent journals that carry a high proportion of observational clinical studies, relative to the number of randomised trials or laboratory studies: *Pediatrics*, *Neurology* and *BMJ Open*.

We categorised articles published in the (arbitrarily) selected month according to the following questions:

1. Was any form of univariable or multivariable regression analysis reported? (Y/N)
2. If yes to q.1, what was the type of research question?
   (D = descriptive, P = predictive, C = causal, V = vague or imprecisely stated)
3. For articles reporting the results of regression analysis (all Y/N):
   1. If the question was descriptive, was multivariable regression used without a sensible rationale? (For example, for comparisons between subpopulations, was regression used to adjust for covariates without a clear reason? Sensible rationale would mention the use of regression as a tool for smoothing or variance reduction, or standardisation, and not describe regression as controlling or adjusting for a covariate as if it were a confounder.)
   2. If the question was predictive,
      1. Did the development of an appropriate regression model omit consideration of predictive performance (validation) as a criterion for appropriateness of the model?
      2. Were coefficient estimates interpreted as providing an indication of the “strength of effect” of the corresponding predictors?
   3. If the question was causal,
      1. Were there issues in adjustment for confounding: either not done or not using an appropriate method to identify a set of variables that needed to be controlled or adjusted for? (Appropriate methods involve consideration of causal assumptions based on substantive knowledge; inappropriate methods omit any justification for the adjustment set or use statistical tests including “variable selection” in multivariable regression.)
      2. Was there evidence of the “Table 2 fallacy”, i.e. interpretation of the coefficients of adjustment covariates as if they represented a causal effect of their own?
   4. If the question was vague,
      1. Were the authors using multivariable regression to identify “important risk factors” (or similar)?
      2. Was there evidence of the “Table 2 fallacy”, i.e. interpretation of the coefficients of covariates in the regression as if they represented causal effects, perhaps in the vein of “interesting” (implicitly causal) associations?

**Results**

For each of the three journals, we present a summary table displaying the breakdown of papers reviewed according to the four categories of research purpose, showing within each category the number of articles found to exhibit at least one of the misuses of regression analysis listed in the Methods. The tables give a brief description of the types of misuse identified; further details can be found in notes provided on each article, which follow a full listing in Table A2.5 of all articles and their classification on the review questions (linked by consecutive “study identifiers” assigned to each article within each journal).

**Table A2.1**: Papers using regression analysis (11 of a total of 18 reviewed) in the journal *Pediatrics* (Vol.149, Issue 6, June 2022), classified according to the purpose (type of research question) underlying the analysis.

| **Type of research question** | **Descriptive** | **Predictive** | **Causal** | **Vague/unclear** | **Total** |
| --- | --- | --- | --- | --- | --- |
| ***n*** | 2 | 0 | 6 | 3 | 11 |
| **Problems found** | 1  (no justification for adjustment) |  | 4  (1 univariable, ignoring confounding,  1 unclear about confounding adjustment,  2 problems in method used to identify adjustment set) | 3  (all risk factor identification & version of T2 fallacy) | 8 |
| **Study identifiers** (sequential numbering from Table A2.5) | | | | | |
| Misuse: yes  Misuse: no | P16  P3 | -  - | P4,P5,P8,P9  P1,P6 | -  P14,P15,P17 |  |

**Table A2.2**: Papers using regression analysis (13 of a total of 19 reviewed) in the journal *Neurology* (Vol.98, Issues 23 and 24, June 2022), classified according to the purpose (type of research question: D = descriptive, P = predictive, C = causal, V = vague) underlying the analysis.

| **Type of research question** | **Descriptive** | **Predictive** | **Causal** | **Vague/ unclear** | **Total** |
| --- | --- | --- | --- | --- | --- |
| ***n*** | 1 | 6 | 6 | 0 | 13 |
| **Problems found** | 1  (no justification for adjustment) | 3  (3 inappropriate model dev, 1 also interpretation of coeffs) | 4  (3 problems in method used to identify adjustment set,  1 Table 2 fallacy) |  | 8 |
| **Study identifiers** (sequential numbering from Table A2.5) | | | | | |
| Misuse: yes  Misuse: no | N14  - | N1,N12,N5  N2,N13,N19 | N11,N15,N16,N9  N4,N17 | -  - |  |

**Table A2.3**: Papers using regression analysis (12 of a total of 20 reviewed) in the journal *BMJ Open* (Vol.12, Issue 6, June 2022: the first 20 articles published), classified according to the purpose (type of research question: D = descriptive, P = predictive, C = causal, V = vague) underlying the analysis.

| **Type of research question** | **Descriptive** | **Predictive** | **Causal** | **Vague/unclear** | **Total** |
| --- | --- | --- | --- | --- | --- |
| ***n*** | 2 | 1 | 2 | 7 | 12 |
| **Problems found** | 1  (no justification for adjustment) | 1  (interpretation of coeffs) | 0 | 7  (risk factor identification & version of T2 fallacy) | 9 |
| **Study identifiers** (sequential numbering from Table A2.5) | | | | | |
| Misuse: yes  Misuse: no | B4  B2 | B8  - | -  B7,B20 | B1,B3,B5,B12,B15,B17,B18  - |  |

**Summary**

Overall, we examined 57 papers, in 36 (63%) of which regression methods were used. Among these papers, 25 (69%, or 44% of all papers) exhibited a type of misuse of regression along the lines we have described. Although the mix of types of question was quite different across the three journals (see Table A2.4), the proportion of papers in which misuse could be identified was similar.

**Table A2.4**: Summary of review of clinical research papers focusing on those using regression methods, classified according to the purpose (type of research question: D = descriptive, P = predictive, C = causal, V = vague) underlying the analysis. Values shown are number of articles displaying misuse / total number using regression analysis.

|  | *Pediatrics n* = 18 | *Neurology  n* = 19 | *BMJ Open  n* = 20 | Combined *n* = 57 |
| --- | --- | --- | --- | --- |
| **Descriptive** | 1/2 | 1/1 | 1/2 | 3/5 |
| **Predictive** | - | 3/6 | 1/1 | 4/7 |
| **Causal** | 4/6 | 4/6 | 0/2 | 8/14 |
| **Vague/unclear** | 3/3 | - | 7/7 | 10/10 |
| **Overall (proportion)** | 8/11 (73%) | 8/13 (62%) | 9/12 (75%) | 25/36 (69%) |
| **Proportion all articles** | 44% | 42% | 45% | 44% |

The major problems identified with the use of regression analysis were:

- 10 papers in which regression was used to answer a poorly specified question, along the lines of “we aimed to identify the important risk factors for *Y*”, and results were almost universally presented as a table of estimated coefficients for a multivariable regression model, with these interpreted as providing the “independent effect” of the corresponding factor on the outcome.
- 8 papers that used outcome regression analysis to answer a clearly specified causal question (or questions) but failed to provide a clear rationale for the choice of adjustment variables needed to control for confounding (or failed to acknowledge the risk of confounding bias), and in one case interpreted coefficients of a multivariable model as causal effects.
- 4 papers with the objective of developing a prediction model, among which 3 failed to use an appropriate method for developing their model (i.e. based this on statistical significance of terms in the regression model rather than impact on predictive validity) and 2 interpreted coefficients in a pseudo-causal fashion.
- 3 papers using regression for a descriptive research purpose but including adjustment terms without clear justification.

**Table A2.5: Full listing of all articles reviewed, with coding according to review questions (see Methods)**

Coding: Y=yes, N=no, D = descriptive, P = predictive, C = causal, V = vague or imprecisely stated.

| ***ID no.*** | ***Article*** | ***Q1*** | ***Q2*** | ***Q3a*** | ***Q3b(i)*** | ***Q3b(ii)*** | ***Q3c(i)*** | ***Q3c(ii)*** | ***Q3d(i)*** | ***Q3d(ii)*** |
| --- | --- | --- | --- | --- | --- | --- | --- | --- | --- | --- |
|  | ***Pediatrics*** |  |  |  |  |  |  |  |  |  |
| P1. | Whitaker RC, et al. Family Connection and Flourishing Among Adolescents in 26 Countries. *Pediatrics.* 2022;149(6). | Y | C | - | - | - | N | N | - | - |
| P2. | Sharko M, et al. State-by-State Variability in Adolescent Privacy Laws. *Pediatrics.* 2022;149(6). | N | D |  |  |  |  |  |  |  |
| P3. | Rahman R, et al. Intimate Partner Violence and the COVID-19 Pandemic. *Pediatrics.* 2022;149(6). | Y | D | N |  |  |  |  |  |  |
| P4. | Rao S, et al. Asthma and the Risk of SARS-CoV-2 Infection Among Children and Adolescents. *Pediatrics.* 2022;149(6). | Y | C | - | - | - | Y | N | - | - |
| P5. | Boutzoukas AE, et al. School Masking Policies and Secondary SARS-CoV-2 Transmission. *Pediatrics.* 2022;149(6). | Y | C | - |  |  | Y | N |  |  |
| P6. | Elliott LJ, et al. Vegetarian Diet, Growth, and Nutrition in Early Childhood: A Longitudinal Cohort Study. *Pediatrics.* 2022;149(6). | Y | C | - | - | - | N | N | - | - |
| P7. | Morin L, et al. The Current and Future State of Pediatric Sepsis Definitions: An International Survey. *Pediatrics.* 2022;149(6). | N | D |  |  |  |  |  |  |  |
| P8. | Oghalai JS, et al. Cochlear Implants for Deaf Children With Early Developmental Impairment. *Pediatrics.* 2022;149(6). | Y | C | - | - | - | Y | N | - | - |
| P9. | Boyle MH, et al. Physical Activity Opportunities in US Early Child Care Programs. *Pediatrics.* 2022;149(6). | Y | D/C | - | - | - | Y | N | - | - |
| P10. | Khan A, et al. Family Safety Reporting in Medically Complex Children: Parent, Staff, and Leader Perspectives. *Pediatrics.* 2022;149(6). | N | D |  |  |  |  |  |  |  |
| P11. | Guez-Barber D, et al. Differentiating Bell’s Palsy From Lyme-Related Facial Palsy. *Pediatrics.* 2022;149(6). | N | D/P |  |  |  |  |  |  |  |
| P12. | Liao F-M, et al. Direct Bilirubin and Risk of Biliary Atresia. *Pediatrics.* 2022;149(6). | N | D |  |  |  |  |  |  |  |
| P13. | Gupta K, et al. Dextrose Gel for Neonates at Risk With Asymptomatic Hypoglycemia: A Randomized Clinical Trial. *Pediatrics.* 2022;149(6). | N | C |  |  |  |  |  |  |  |
| P14. | Boghossian NS, et al. Transfer Patterns of Very Low Birth Weight Infants for Convalescent Care. *Pediatrics.* 2022;149(6). | Y | V | - | - | - | - | - | Y | Y |
| P15. | Aubert AM, et al. Movement Difficulties at Age Five Among Extremely Preterm Infants. *Pediatrics.* 2022;149(6). | Y | D/V | - | - | - | - | - | Y | Y |
| P16. | de Almeida MFB, et al. Translating Neonatal Resuscitation Guidelines Into Practice in Brazil. *Pediatrics.* 2022;149(6). | Y | D | Y | - | - | - | - | - | - |
| P17. | Price JJ, et al. Cardiovascular Risk Factors and Target Organ Damage in Adolescents: The SHIP AHOY Study. *Pediatrics.* 2022;149(6). | Y | V | - | - | - | - | - | Y | Y |
| P18. | Prins S, et al. How Physicians Discuss Uncertainty With Parents in Intensive Care Units. *Pediatrics.* 2022;149(6). | N | D |  |  |  |  |  |  |  |
|  |  |  |  |  |  |  |  |  |  |  |
|  | ***Neurology*** |  |  |  |  |  |  |  |  |  |
| N1. | Faizy TD, et al. The Cerebral Collateral Cascade. *Comprehensive Blood Flow in Ischemic Stroke.* 2022;98(23):e2296-e2306. | Y | P | - | Y | Y | - | - | - | - |
| N2. | Yang S, et al. Development of a Model to Predict 10-Year Risk of Ischemic and Hemorrhagic Stroke and Ischemic Heart Disease Using the China Kadoorie Biobank. *Neurology.* 2022;98(23):e2307-e2317. | Y | P | - | N | N | - | - | - | - |
| N3. | Stefanetti RJ, et al. L-Arginine in Mitochondrial Encephalopathy, Lactic Acidosis, and Stroke-like Episodes. *A Systematic Review.* 2022;98(23):e2318-e2328. | N |  |  |  |  |  |  |  |  |
| N4. | Wiggs KK, et al. Maternal Serotonergic Antidepressant Use in Pregnancy and Risk of Seizures in Children. *Neurology.* 2022;98(23):e2329-e2336. | Y | C | - | - | - | N | N | - | - |
| N5. | Gross WL, et al. Prediction of Naming Outcome With fMRI Language Lateralization in Left Temporal Epilepsy Surgery. *Neurology.* 2022;98(23):e2337-e2346. | Y | P | - | Y | N | - | - | - | - |
| N6. | Morris C, et al. Outcomes That Matter to Adolescents With Continuous Headache Due to Chronic Migraine and Their Parents. *A Pilot Survey Study.* 2022;98(23):e2347-e2355. | N |  |  |  |  |  |  |  |  |
| N7. | Thomas FP, et al. Randomized Phase 2 Study of ACE-083 in Patients With Charcot-Marie-Tooth Disease. *Neurology.* 2022;98(23):e2356-e2367. | N |  |  |  |  |  |  |  |  |
| N8. | Molimard A, et al. Rituximab Therapy in the Treatment of Juvenile Myasthenia Gravis. *The French Experience.* 2022;98(23):e2368-e2376. | N |  |  |  |  |  |  |  |  |
| N9. | Raj R, et al. Risk of Dementia After Hospitalization Due to Traumatic Brain Injury. *A Longitudinal Population-Based Study.* 2022;98(23):e2377-e2386. | Y | C | - | - | - | N | Y | - | - |
| N10. | Gool JK, et al. Data-Driven Phenotyping of Central Disorders of Hypersomnolence With Unsupervised Clustering. *Neurology.* 2022;98(23):e2387-e2400. | N |  |  |  |  |  |  |  |  |
| N11. | Vitkova M, et al. Association of Latitude and Exposure to Ultraviolet B Radiation With Severity of Multiple Sclerosis. *An International Registry Study.* 2022;98(24):e2401-e2412. | Y | D/C | Y |  |  | Y | N | - | - |
| N12. | Yoon EJ, et al. Brain Metabolism Related to Mild Cognitive Impairment and Phenoconversion in Patients With Isolated REM Sleep Behavior Disorder. *Neurology.* 2022;98(24):e2413-e2424. | Y | D/P | Y | Y | Y | - | - | - | - |
| N13. | Petersen KK, et al. Predicting Amyloid Positivity in Cognitively Unimpaired Older Adults. *A Machine Learning Approach Using A4 Data.* 2022;98(24):e2425-e2435. | Y | P | - | N | N | - | - | - | - |
| N14. | Whitwell JL, et al. Investigating Heterogeneity and Neuroanatomic Correlates of Longitudinal Clinical Decline in Atypical Alzheimer Disease. *Neurology.* 2022;98(24):e2436-e2445. | Y | D | Y | - | - | - | - | - | - |
| N15. | Sible IJ, et al. Visit-to-Visit Blood Pressure Variability and CSF Alzheimer Disease Biomarkers in Cognitively Unimpaired and Mildly Impaired Older Adults. *Neurology.* 2022;98(24):e2446-e2453. | Y | C | - | - | - | Y | N | - | - |
| N16. | Tarko L, et al. Racial and Ethnic Differences in Short- and Long-term Mortality by Stroke Type. *Neurology.* 2022;98(24):e2465-e2473. | Y | C | - | - | - | Y | N | - | - |
| N17. | Cai M, et al. Association of Ambient Particulate Matter Pollution of Different Sizes With In-Hospital Case Fatality Among Stroke Patients in China. *Neurology.* 2022;98(24):e2474-e2486. | Y | C | - | - | - | N | N | - | - |
| N18. | Grindegård L, et al. Association Between EEG Patterns and Serum Neurofilament Light After Cardiac Arrest. *A Post Hoc Analysis of the TTM Trial.* 2022;98(24):e2487-e2498. | N |  |  |  |  |  |  |  |  |
| N19. | Abdallah C, et al. Clinical Yield of Electromagnetic Source Imaging and Hemodynamic Responses in Epilepsy. *Validation With Intracerebral Data.* 2022;98(24):e2499-e2511. | Y | P | - | N | N | - | - | - | - |
|  |  |  |  |  |  |  |  |  |  |  |
|  | ***BMJ Open*** |  |  |  |  |  |  |  |  |  |
| B1. | Cullen P, et al. Returning to the emergency department: a retrospective analysis of mental health re-presentations among young people in New South Wales, Australia. *BMJ Open.* 2022;12(6):e057388. | Y | P/V | - | - | - | - | - | Y | Y |
| B2. | Gardner LA, et al. Lifestyle risk behaviours among adolescents: a two-year longitudinal study of the impact of the COVID-19 pandemic. *BMJ Open.* 2022;12(6):e060309. | Y | D | N | - | - | - | - | - | - |
| B3. | Bradfield OM, et al. Vocational and psychosocial predictors of medical negligence claims among Australian doctors: a prospective cohort analysis of the MABEL survey. *BMJ Open.* 2022;12(6):e055432. | Y | V | - | - | - | - | - | Y | Y |
| B4. | Rogers A, et al. Adverse events and overall health and well-being after COVID-19 vaccination: interim results from the VAC4COVID cohort safety study. *BMJ Open.* 2022;12(6):e060583. | Y | D | Y | - | - | - | - | - | - |
| B5. | Ha TN, et al. Trend in CT utilisation and its impact on length of stay, readmission and hospital mortality in Western Australia tertiary hospitals: an analysis of linked administrative data 2003–2015. *BMJ Open.* 2022;12(6):e059242. | Y | V | - | - | - | - | - | Y | Y |
| B6. | Alharbi K, et al. Understanding the implementation of interventions to improve the management of frailty in primary care: a rapid realist review. *BMJ Open.* 2022;12(6):e054780. | N |  |  |  |  |  |  |  |  |
| B7. | Vijayaraghavan BKT, et al. Hydroxychloroquine plus personal protective equipment versus personal protective equipment alone for the prevention of laboratory-confirmed COVID-19 infections among healthcare workers: a multicentre, parallel-group randomised controlled trial from India. *BMJ Open.* 2022;12(6):e059540. | Y | C | - | - | - | - | - | N | N |
| B8. | Ma Y, et al. Development of a novel predictive model for a successful stone removal after flexible ureteroscopic lithotripsy based on ipsilateral renal function: a single-centre, retrospective cohort study in China. *BMJ Open.* 2022;12(6):e059319. | Y | P | - | N | Y | - | - | - | - |
| B9. | Sania A, et al. Rapid antigen testing by community health workers for detection of SARS-CoV-2 in Dhaka, Bangladesh: a cross-sectional study. *BMJ Open.* 2022;12(6):e060832. | N |  |  |  |  |  |  |  |  |
| B10. | Wilander H, et al. Nationwide observational study of incidence, management and outcome of spontaneous coronary artery dissection: a report from the Swedish Coronary Angiography and Angioplasty register. *BMJ Open.* 2022;12(6):e060949. | N |  |  |  |  |  |  |  |  |
| B11. | Puértolas OC, et al. Evaluating the controlled reopening of nightlife during the COVID-19 pandemic: a matched cohort study in Sitges, Spain, in May 2021 (Reobrim Sitges). *BMJ Open.* 2022;12(6):e058595. | N |  |  |  |  |  |  |  |  |
| B12. | Tiwari L, et al. Clinicodemographic profile and predictors of poor outcome in hospitalised COVID-19 patients: a single-centre, retrospective cohort study from India. *BMJ Open.* 2022;12(6):e056464. | Y | V | - | - | - | - | - | Y | Y |
| B13. | Bywall KS, et al. Making space for patients’ preferences in precision medicine: a qualitative study exploring perspectives of patients with rheumatoid arthritis. *BMJ Open.* 2022;12(6):e058303. | N |  |  |  |  |  |  |  |  |
| B14. | Lurie P, et al. COVID-19 vaccine misinformation in English-language news media: retrospective cohort study. *BMJ Open.* 2022;12(6):e058956. | N |  |  |  |  |  |  |  |  |
| B15. | Muhammad T, et al. Socioeconomic and health-related inequalities in major depressive symptoms among older adults: a Wagstaff’s decomposition analysis of data from the LASI baseline survey, 2017–2018. *BMJ Open.* 2022;12(6):e054730. | Y | V | - | - | - | - | - | Y | Y |
| B16. | Moussallem M, et al. Evaluating the governance and preparedness of the Lebanese health system for the COVID-19 pandemic: a qualitative study. *BMJ Open.* 2022;12(6):e058622. | N |  |  |  |  |  |  |  |  |
| B17. | Silwal PR, et al. Understanding geographical variations in health system performance: a population-based study on preventable childhood hospitalisations. *BMJ Open.* 2022;12(6):e052209. | Y | V | - | - | - | - | - | Y | Y |
| B18. | Silver NA, et al. Charming e-cigarette users with distorted science: a survey examining social media platform use, nicotine-related misinformation and attitudes towards the tobacco industry. *BMJ Open.* 2022;12(6):e057027. | Y | V | - | - | - | - | - | Y | Y |
| B19. | Yu H, et al. RECIST 1.1 versus mRECIST for assessment of tumour response to molecular targeted therapies and disease outcomes in patients with hepatocellular carcinoma: a systematic review and meta-analysis. *BMJ Open.* 2022;12(6):e052294. | N |  |  |  |  |  |  |  |  |
| B20. | Adhikari I, et al. Association of *Chlamydia trachomatis* infection with cervical atypia in adolescent women with short-term or long-term use of oral contraceptives: a longitudinal study in HPV vaccinated women. *BMJ Open.* 2022;12(6):e056824. | Y | C | - | - | - | N | N | - | - |

**Notes on articles reviewed, to support coding according to review questions**

P3. Paper documents an increase in intimate partner violence referrals after the start of the COVID-19 pandemic. Description of “before” and “after” with a univariable regression used to assess the size of change between periods.

P4. Examines whether asthma is associated with increased risk of COVID-19. Uses propensity score matching: “Because we identified substantial differences in patient characteristics and SARS-CoV-2 testing rates by asthma status, we used propensity score matching to construct a cohort of children with asthma and control children without asthma closely matched on these variables.” Don’t mention confounding as such and not clear about the need for the variables used to account for all confounding between asthma status and risk of COVID. (Y,N)

P5. District-level analysis of incidence of SARS-CoV-2 infections, comparing 3 masking policy regimes. No mention of potential confounding. Regression seems to have been used only for unadjusted comparisons. (Y,N)

P6. Focus on causal effect of “vegetarian diet” with multiple adjustments “determined a priori from literature review”.

P8. Aim was to evaluate the effect of cochlear implants by comparing 3 cohorts who had differential access to this treatment. No adjustments were made for potential confounding, on the basis that they “found no differences among the cohorts on the basis of study site, sex, household income, race, or mother’s education level (Table 1).” Regression used to estimate “trajectories” in outcomes over time allowing for interaction of cohort with age. (Y,N)

P9. First part of paper is descriptive analysis of physical activity in early childhood education centres. Final part investigates the (causal) effects of potential “barriers to physical activity” using regression to adjust for covariates. No explicit reason given for chosen list of adjustment variables nor consideration of potential unmeasured confounding. (Y,N)

P13. RCT with no adjustment.

P14. Aims and design were unclear. A large part of the analysis focuses on descriptive presentation, but they use multiple regression to identify “risk factors” with Figure 2 apparently a version of the Table 2 fallacy. Many “significant” risk factors but no mention of possibility of interactions.

P15. Initial aim was to describe prevalence of different levels of disability in a cohort of children with cerebral palsy. Second part “aimed […] to identify sociodemographic, perinatal, and neonatal risk factors associated with movement difficulties.” (Intro) “We produced 3 models to measure the association of sociodemographic, perinatal, and neonatal variables with the probability of being at risk or having significant movement difficulties using multinomial logistic regression…” (Methods) Table 3 exhibits Table 2 fallacy.

P16. Aim was to describe the uptake over time (2014-2020) of best-practice guidelines for neonatal care of preterm newborns (23-31 weeks gestation). Slightly unclear but logistic regression used to estimate change (as OR) per year in use of specific procedures with adjustment for “center and year” (confusing because year seemed to be the factor of interest in the analysis).

P17. “To address the primary research question, generalized linear models were constructed to determine if CVRFs in combination with other known risk factors of end organ changes, including demographic, biometric and laboratory analyses, could predict TOD in our population.” Despite this wording, the primary interest appeared to be causal (last sentence of Conclusion: “Future studies should address whether amelioration of these CVRFs in the young averts development of TOD and adult cardiovascular disease.”) N.B. Backward elimination was used to determine adjustment set.

N1. Cohort study looking at prediction or risk stratification according to “CCC” status. “Primary outcome analysis” m-v binary logistic regression model with CCC status (3 levels) adjusted for covariates selected by backwards elimination. Adjusted ORs presented as indicating strength of prediction.

N4. Excellent example of causal inference using outcome regression.

N5. Aimed to determine whether fMRI measure predicted outcome. Main result was that “a hierarchical multivariable regression model showed that fMRI added significant independent predictive value beyond the other predictors” (not clear what “hierarchical” meant here), based on first reducing covariates according to “significance” and then comparing R-sq before and after adding fMRI. Small sample size n=81.

N9. Primary analysis focused on causal effect of TBI on risk of dementia with appropriate adjustment for confounders, but results presented in Table 2 and Figure 3 exhibit “Table 2 fallacy”.

N11. Aimed to determine the “effect” of latitude on multiple sclerosis severity. Main analysis was regression adjusted for “confounders” but no discussion of what this meant or why this adjustment set was chosen. If aim descriptive then adjustment unwarranted, but if aim causal (latitude as a proxy for some possible intervention?) then choice of adjustments seemed odd because most of them followed the “exposure” to latitude.

N12. Aimed to stratify patients with rare disorder according to risk of progression to Parkinson’s or dementia. Used Cox regression for four putative predictors, 3 selected by screening of image-based measures of brain glucose metabolism, adjusted for age and disease duration. Meaning of adjusted HRs?

N13. Prediction modelling using machine learning.

N14. Describing patterns of clinical assessment measures for patients with two neurological diseases, using “linear mixed-effects model” apparently to estimate mean at baseline and mean rate of change. Adjustment for age at baseline not explained.

N15. “Bayesian linear growth modeling with the brms package […] in R investigated the role of BPV, APOE ε4, and the passage of time on CSF AD biomarker levels.” Various interactions and adjustments with unclear rationale.

N16. Effects of race on mortality – arguably a causal question, with authors interested in finding explanations for the disparities, but choice of adjustment variables unclear and included “post-exposure” variables such as comorbidities.

N17. Continuous exposure, attempted confounder adjustment.

N19. Development of MRI measures to predict postsurgical outcome in epilepsy (small sample, informal prediction). Simple univariable regression used in describing results.

B1. Aims were to identify “key characteristics associated with higher risk of […] re-presentation.” Used both univariate and m-v regression, concluding that the latter “resulted in the same set of significantly associated characteristics and did not change the observations and interpretations made with the univariate regression analyses” (?)

B2. Comparing health-related behaviours in a cohort before and during the pandemic. Used GLMMs to estimate prevalence ratios (random effects for student and school) with no covariate adjustment. Some acknowledgement of potential confounding if looking for causal interpretation.

B3. Multivariate logistic regression for risk of “medical negligence”. “Our multivariate analysis identified a number of significant demographic risk factors associated with being sued”. Discussion exhibits Table 2 fallacy.

B4. Descriptive analysis with K-M estimation of proportions over time, but Cox regression used to compare between vaccines adjusted for age, with no explanation for adjustment.

B5. Two major analyses: (1) multivariable Poisson regression models used “to identify factors associated with each classification of CT use” – see Table 2 (fallacy); (2) compared outcomes between CT exposure groups over time using some form of g-computation (predictive margins)

B8. Made some use of cross-validation in setting tuning parameter for LASSO, and mentioned AUC and calibration, but Table 2 and related text presented coefficient estimates as indicating strength of “independent prediction”

B12. “Binary logistic regression was done to identify predictors of mortality, and multivariable regression analysis was done to control potential confounders.” Table 4 exhibits Table 2 fallacy.

B15. “In this study, […] objective of finding the association between socioeconomic and health status and depression among older adults and explore the contributing factors in the inequalities in late-life depression”. M-v logistic regression presented in Table 3 and interpreted as showing factors “significantly associated with” outcome. Also combined regression coefficients to produce “Wagstaff’s Concentration Index” (?).

B17. Multilevel logistic regressions used – combination of trying to quantify geographic variability (adjusted…) along with determining “effects” of “key sociodemographic, economic, geographical and health system characteristics”. See Table 4 and text referring to for version of Table 2 fallacy.

B18. Using m-v regression models to investigate “the extent to which use of specific social media platforms are associated with [outcomes]”. Tables 2 and 3 appear to present “mutually adjusted” coefficient estimates, interpreted in the text as causal effects.
